# Supplementary material for: An examination of the Devonian fishes of Michigan
Source: PeerJ. 2018 Sep 20;6:e5636. doi: 10.7717/peerj.5636 (PMC6151260; doi:10.7717/peerj.5636)
Supplement: Table S1 [file peerj-06-5636-s001.docx]

| **Locality** | Sibley Quarry | Trenton | Monroe County | Rogers City | Rockport Quarry |
| --- | --- | --- | --- | --- | --- |
| **Vertebrates** | ?*Titanichthys* sp., *Onychodus sigmoides,* and *Acondylacanthus gracillimus* | ?*Macropetalichthys* sp. and *Ptyctodus* sp. | *Onychodus* sp. and *Machaeracanthus* sp. | *Ptyctodus* sp. | *Protitanichthys rockportensis*, ?*Mylostoma* sp., ?*Holonema rugosum*, *Dinomylostoma* sp., *Dunkleosteus* sp.*, Mylostoma* sp., *Ptyctodus* sp., ?*Tamiobatis* sp., and ?*Machaeracanthus* sp. |
| **International** **Stage** | Eifelian | Eifelian | Eifelian | Givetian | Givetian |
| **Regional Stage** | Ulsterian | Ulsterian | Ulsterian | Early Erian | Early Erian |
| **Formation** | Dundee Limestone | Dundee Limestone | Dundee Limestone | Bell Shale | Rockport Quarry Limestone. |
| **County** | Wayne | Wayne | Monroe | Presque Isle | Alpena |
| **City** | Trenton | Trenton | Unknown | Rogers City | Alpena |
| **Location** | Sibley Quarry near Fort Street and Sibley Road, Wyandotte, Wayne County | Near Trenton | Unknown | Unknown | NE Michigan. Abandoned strip mine in Rockport State Park 15 miles north of Alpena. |
