# Supplementary material for: An examination of the Devonian fishes of Michigan
Source: PeerJ. 2018 Sep 20;6:e5636. doi: 10.7717/peerj.5636 (PMC6151260; doi:10.7717/peerj.5636)
Supplement: Table S2 [file peerj-06-5636-s002.docx]

| **Locality** | French Road | Posen | Onaway Stone Quarry | South Point | Besser Museum Fossil Park |
| --- | --- | --- | --- | --- | --- |
| **Vertebrates** | ?*Holonema rugosum* | ?*Eczematolepis* sp. | *Holonema farrowi*, *Machaeracanthus* sp., and *Chirodipterus onawayensis* | ?*Onychodus* sp., *Gyracanthus* sp., and an unidentified holonemiid | ?*Mylostoma* sp. and  ?*Macropetali-*  *thys* sp. |
| **International** **Stage** | Givetian | Givetian | Givetian | Givetian | Givetian |
| **Regional Stage** | Early Erian | Early Erian | Middle Erian | Middle Erian | Middle Erian |
| **Formation** | Genshaw Formation | Genshaw Formation | Newton Creek Limestone | Gravel Point (South Point) | Alpena Limestone |
| **County** | Alpena | Presque Isle | Presque Isle | Charlevoix | Alpena |
| **City** | Near Alpena | Near Posen | Onaway | South Point | Alpena |
| **Location** | French Road near Long Lake, near Rockport Quarry, Alpena County. | Near Posen. | Onaway Stone Quarry, north edge of Onaway, Presque Isle County. | Exposures along Lake Michigan shore at South Point, little Traverse Bay. | Fossil park maintained by the Besser Museum in Alpena, Michigan. |
