# Supplementary material for: An examination of the Devonian fishes of Michigan
Source: PeerJ. 2018 Sep 20;6:e5636. doi: 10.7717/peerj.5636 (PMC6151260; doi:10.7717/peerj.5636)
Supplement: Table S3 [file peerj-06-5636-s003.docx]

| **Locality** | Alkali Quarry | Betsie Bay Rockpiles | Four Mile Dam | Old Wamer’s Brickyard | Alpena Cementary |
| --- | --- | --- | --- | --- | --- |
| **Vertebrates** | *Ptyctodus* sp. and *Dunkleosteus* sp. | ?*Mylostoma* sp., ?*Macropetalichthys* sp., *Protitanichthys rockportensis*, an unidentified acanthodian, and an unidentified placoderm | *Oracanthus* sp. | *Ptyctodus* sp. | *Ptyctodus* sp. |
| **International** **Stage** | Givetian | Givetian | Givetian | Givetian | Givetian |
| **Regional Stage** | Middle Erian | Late Erian | Late Erian | Late Erian | Late Erian |
| **Formation** | Alpena Limestone | Four Mile Dam | Norway Point | Potter Farm? | Potter Farm |
| **County** | Alpena | Benzie | Alpena | Alpena | Alpena |
| **City** | Alpena | Elberta | Unknown | Alpena | Alpena |
| **Location** | Alkali Quarry, loose block. | NW Michigan: Southern Shore of Betsie Lake near the Bay: Village of Elberta: adjacent to Waterfront Park: Village of Elberta: Bruce Tobin property. Rocks at the site are originally from the Specification Stone Products Quarry in Alpena, MI. Address: 1009 Long Lake Rd, Alpena, MI 49707. | Four Mile Dam about 3.5 miles northwest of Alpena. | Southwest of Alpena | West edge of Alpena Cemetery, Evergreen Cemetery. |
