# Supplementary material for: An examination of the Devonian fishes of Michigan
Source: PeerJ. 2018 Sep 20;6:e5636. doi: 10.7717/peerj.5636 (PMC6151260; doi:10.7717/peerj.5636)
Supplement: Table S4 [file peerj-06-5636-s004.docx]

| **Locality** | Partridge Point | Norwood | Squaw Bay | Grand Traverse Bay | Paxton Quarry |
| --- | --- | --- | --- | --- | --- |
| **Vertebrates** | *Ptyctodus* sp. | ?*Trachosteus clarki* | *Dunkleosteus* sp. | *Aspidicthys clavatus* | *Diplognathus lafargei* |
| **International Stage** | Givetian | Frasnian/Famennian | Frasnian/Famen-nian | Frasnian/Famennian | Frasnian/Famenni-an |
| **Regional Stage** | Late Erian | Senecan/Chatauqu-an | Senecan/Chat-auquan | Senecan/Chatauqu-an | Senecan/Chatauq-uan |
| **Formation** | Thunder Bay Limestone | Antrim Shale | Antrim Shale | Antrim Shale | Antrim Shale |
| **County** | Alpena | Charlevoix | Alpena | Charlevoix | Alpena |
| **City** | Unknown | Norwood | Alpena | Norwood | Alpena |
| **Location** | Bluffs on the northeast shore of Partridge Point, 6.4 kilometers south of Alpena. | 1.6 kilometers north of Norwood. Exact location unknown. | Squaw Bay, 6.4 kilometers south of Alpena on U.S 23. | Shore of Grand Traverse Bay near Norwood. Exact location unknown. | Paxton Quarry (Lafarge North America, Inc., Alpena Cement Plant, Great Lakes Region), Alpena. |
