# Supplementary material for: An examination of the Devonian fishes of Michigan
Source: PeerJ. 2018 Sep 20;6:e5636. doi: 10.7717/peerj.5636 (PMC6151260; doi:10.7717/peerj.5636)
Supplement: Supplemental Information 1 [file peerj-06-5636-s005.docx]

Dundee Limestone Formation

Acanthodii

Ischnacanthiformes

Machaeracanthidae

*Machaeracanthus* sp.

Chondrichthyes

Ctenacanthiformes

Ctenacanthoidea incertae sedis

*Acondylacanthus gracillimus*

Placodermi

Arthrodira

Titanichthyidae

*Titanichthys*? sp.

Petalichthyida

*Macropetalichthys* sp.

Sarcopterygii

Onychodontiformes

Onychodontidae

*Onychodus sigmoides*

Rogers City Limestone Formation

Acanthodii

Climatiida

Gyracanthidae

*Gyracanthus* sp.

Bell Shale

Placodermi

Ptyctodontida

Ptyctodontidae

*Ptyctodus* sp.

Rockport Quarry Limestone

Placodermi

gen. et. sp. indet.

Arthrodira

Coccosteoidea

*Protitanicthys rockportensis*

Dinichthyidae

*Dunkleosteus* sp.

Holonematidae

*Holonema rugosum*?

*Holonema* sp.

Mylostomatidae

*Mylostoma* sp.

*Mylostoma*? sp.

*Dinomylostoma* sp.

Ptyctodontida

Ptyctodontidae

*Ptyctodus* sp.

Chondrichthyes

Ctenacanthiformes

Tamiobatidae

*Tamiobatis*? sp.

Acanthodii

Ischnacanthiformes

Machaeracanthidae

*Machaeracanthus*? sp.

Genshaw Formation

Placodermi

Ptyctodontida

Ptyctodontidae

*Eczematolepis*? sp.

Arthrodira

Holonematidae

*Holonema rugosum*?

Newton Creek Formation

Placodermi

Arthrodira

Holonematidae

*Holonema farrowi*

Sarcopterygii

Dipnoiformes

Dipteridae

*Chirodipterus onawayensis*

Acanthodii

Ischnacanthiformes

Machaeracanthidae

*Machaeracanthus* sp.

Gravel Point Formation

Acanthodii

Climatiida

Gyracanthidae

*Gyracanthus* sp.

Sarcopterygii

Onychodontiformes

Onychodontidae

*Onychodus*? sp.

Placodermi

Arthrodira

Holonematidae

Holonemiid

Alpena Limestone Formation

Placodermi

Arthrodira

Mylostomatidae

*Mylostoma*? sp.

Dinichthyidae

*Dunkleosteus* sp.

Petalichthyida

*Macropetalichthys*? sp.

Ptyctodontida

Ptyctodontidae

*Ptyctodus* sp.

Four Mile Dam Formation

Placodermi

gen. et. sp. indet.

Arthrodira

Coccosteoidea

*Protitanicthys rockportensis*

Mylostomatidae

*Mylostoma*? sp.

Petalichthyida

*Macropetalichthys*? sp.

Acanthodii

gen. et. sp. indet.

Ischnacanthiformes

Machaeracanthidae

*Machaeracanthus*? sp.

Norway Point Formation

Acanthodii

Climatiida

Gyracanthidae

*Oracanthus* sp.

Potter Farm Formation

Placodermi

Ptyctodontida

Ptyctodontidae

*Ptyctodus* sp.

Thunder Bay Limestone

Placodermi

Ptyctodontida

Ptyctodontidae

*Ptyctodus* sp.

Antrim Shale

Placodermi

Arthrodira

Dinichthyidae

*Dunkleosteus* sp.

Insertae sedis

*Diplognathus larfargei*

*Aspidichthys clavatus*

*Trachosteus clarkii*?

Ptyctodontida

gen. et. sp. indet.
